# The effects of the natural enzyme, Pectinex Ultra SP-L, on human cell cultures and bacterial biofilms *in vitro*

**DOI:** 10.1186/s12866-014-0251-1

**Published:** 2014-10-02

**Authors:** Ian P Olwoch, Oppel B W Greeff, Gisela Jooné, Vanessa Steenkamp

**Affiliations:** Department of Pharmacology, Faculty of Health Sciences, University of Pretoria, Private Bag X323, Arcadia, 0007 Pretoria, South Africa

**Keywords:** Antibiotic, Biofilm, Cytotoxicity, Enzyme, HeLa cell, Lymphocyte, Neutrophil, Pectinex, *Pseudomonas aeruginosa*, *Staphylococcus aureus*

## Abstract

**Background:**

Pectinex Ultra SP-L (Pectinex) is a microbial-derived enzyme that is used in the food industry and that has been shown to inhibit bacterial biofilms. It has been suggested that Pectinex may be useful in the management of biofilm-related bacterial infections and therefore warrants further investigation in this regard. The aim of this study was to investigate the cytotoxicity of Pectinex on cervical adenocarcinoma cells (HeLa), lymphocytes and neutrophils. Cell viability and morphology were assessed using an *in vitro* spectrophotometric MTT (3-(4, 5-dimethylthiazol-2-yl)-2, 5-diphenyl tetrazolium bromide) assay and polarization-optical transmitted light differential interference contrast microscopy. This study also investigated the antibacterial and antibiofilm actions of Pectinex, alone and in combination with antibiotics, on standard and clinical cultures of *Staphylococcus aureus* and *Pseudomonas aeruginosa.* Minimum inhibitory (MIC) and bactericidal (MBC) concentrations were determined using *p*-iodo-nitrotetrazolium violet staining of bacterial cultures and regrowth of subcultures. Biofilm biomass and cell viability were quantified spectrophotometrically after staining with crystal violet and MTT.

**Results:**

The IC_50_ (±SEM) of Pectinex was 193.9 (±22.2) PGU/ml for HeLa cells, 383.4 (±81.5) and 629.6 (±62.8) PGU/ml for fMLP-stimulated and non-stimulated lymphocytes respectively, and 245.9 (±9.4) and 529.7 (±40.7) PGU/ml for fMLP-stimulated and non-stimulated neutrophils, respectively. Induced morphological features characteristic of apoptosis and necrosis included cell membrane blebs and vacuolization in HeLa cells, clumping in lymphocytes, as well as shrunken rounded cells, apoptotic bodies and debris in all cultures. Pectinex (7.42 – 950 PGU/ml^−1^) was not bactericidal. In clinical cultures of *Staphylococcus aureus*, co-administration of Pectinex was associated with a 28.0% increase in both the MIC and MBC of amoxicillin-clavulanate. In clinical cultures of *P. aeruginosa*, there was an 89.0% and 92.8% increase in the MIC and MBC of ciprofloxacin, respectively. Pectinex ≤ 118.75 PGU/ml^−1^ and incubation periods ≥ 6 h were associated with increased biomass and cell viability in *S. aureus* or *P. aeruginosa* biofilms.

**Conclusions:**

Pectinex appeared to antagonize the antibacterial effects of amoxicillin-clavulanate and ciprofloxacin and furthermore demonstrated significant cytotoxicity. It was therefore deemed unsuitable for the management of either planktonic or biofilm phenotypes of *S. aureus* or *P. aeruginosa.*

## Background

The alarming worldwide increase in the incidence of bacterial resistance to antimicrobial agents has prompted the urgent need for the development of new drugs and treatment strategies [1-3]. In addressing this problem, scientists acknowledge that bacteria naturally occur as integrated surface bound communities; the biofilm. Bacterial biofilms are dynamic, integrated, heterogeneous (spatial, genetic and physiologic) cell communities, encased in a complex extracellular matrix, that are either surface-bound or free-floating in cohesive cell-to-cell clusters [4,5]. In the biofilm-state, bacteria are more resistant to antibiotics, disinfectants and host immune mechanisms than their planktonic phenotype [6]. However, when dispersed from the biofilm, bacteria revert to the planktonic phenotype with rapid restoration of antibiotic sensitivity [4,7]. Several studies have demonstrated that natural enzymes obtained from animals, plants and microbes (bacteria and fungi), are able to prevent the formation and facilitate the removal of bacterial biofilms, and potentiate the action of co-administered antibiotics *in vitro*. Their actions are mediated through enzymatic degradation of the biofilm matrix. As a treatment strategy, biofilm matrix-degrading enzymes may offer a solution to the management of persistent biofilm-related infections when used either as the sole antimicrobial agents or as adjuncts to conventional antibiotic therapy [8].

Many natural enzymes are readily available and in everyday use as catalysts in numerous domestic and industrial applications, including production of food and beverages, paper, textiles, leather, cosmetics and pharmaceuticals [8,9]. Pectinex Ultra SP-L (Pectinex) is a fungal enzyme complex derived from *Aspergillus acule*atus. The main enzyme in the preparation is polygalacturonase. It is used in the maceration of plant cells for the preparation of mash (puree) foods and the extraction of juices and oils from fruits, vegetables and grain [10]. Pectinex has neither bactericidal nor bacteriostatic activity. Yet, it is known to possess both protease and carbohydrase activity and has been shown to degrade the exopolymeric matrix of *Staphylococcus aureus* and *Pseudomonas aeruginosa*. This has led to the suggestion that the potential antibiofilm effect of Pectinex may be useful in the management of bacterial infections [11,12].

Despite the availability of numerous commercial enzymes, very little about their *in vitro* toxicity is known [13]. In general, they are considered to be non-toxic. Industrial processes use enzymes at low levels after which they are either removed or deactivated from the final product, and the orally ingested residual amounts are likely to be digested and rendered harmless. However, they contain added substances such as preservatives and stabilizers which are a potential problem when enzymes are consumed in food and drink [14]. They may also contain toxins and other metabolites from their source microorganisms which may be harmful [15]. The potential for adverse and toxic effects is of concern to regulatory authorities who have recommended that no new enzyme should be introduced into the food and beverage industry, or that existing enzymes be adapted for new applications, without assessment of their safety [16]. Existing proprietary knowledge of Pectinex toxicity may not be applicable to its “off-label” use as an antimicrobial agent. To our knowledge, no toxicity testing of Pectinex has been performed. Therefore, formal investigation is required before this basic research can translate into clinical experimentation and use. Consideration should be given to the effective antimicrobial concentrations and the effect on the tissues that are exposed [17].

This study investigated the effect of Pectinex as a single agent and also when used in combination with amoxicillin-clavulanate and ciprofloxacin against *S. aureus* and *P. aeruginosa*, respectively. The effect of Pectinex on cervical adenocarcinoma cells, lymphocytes and neutrophils was investigated, as these cell types represent host cells that may be found at the site of an infection. This is the first study to report on the combined effect of antibiotics with Pectinex in bacterial biofilms and on the cytotoxicity of Pectinex in cell cultures.

## Methods

### Test agents

Pectinex (9500 polygalacturonase units per millilitre; PGU ml^−1^) was obtained from Novozymes (Pty) Ltd (Johannesburg, SA), amoxicillin-clavulanate from Sandoz (SA) and ciprofloxacin from Adcock Ingram (SA).

### Cell cultures and maintenance

HeLa cells were obtained from the American Type Culture Collection (ATCC CCL-2). Cells were maintained in Eagle’s minimal essential medium supplemented with 10% foetal bovine serum and 1% penicillin and streptomycin (EMEM+). Once adherent cells reached 70-80% confluency, cells were detached with 0.25% trypsin/versene. The concentration of viable cells was determined by the trypan blue exclusion method [18].

Ethical approval to collect blood from healthy adult volunteers was obtained from the University of Pretoria Ethics Committee (26-08-2009). Written informed consent was obtained from the participants. Lymphocytes and neutrophils were isolated by continuous density-gradient centrifugation over Histopaque 1077 (Sigma-Aldrich, St Louis, Missouri, USA) using a method adapted from Pretlow and Luberoff [19]. Working suspensions of lymphocytes and neutrophils were made by dilution with Roswell Park Memorial Institute Medium (RPMI-1640; Sigma-Aldrich) supplemented with 10% volume of autologous serum and 1% penicillin-streptomycin (RPMI+).

### Cytotoxicity microtitre challenge of cell cultures

Round-bottomed 96-well microtitre plates (USA Scientific Inc., USA) were seeded with 100 μl aliquots of cell suspension (2.5 × 10^4^ cells/ml for HeLa cells; 2 × 10^6^ cells/ml for lymphocytes and neutrophils). Wells which served as experiment blanks were devoid of cells and received 180 μl of culture medium and 20 μl of Pectinex 9500 PGU/ml. The plates seeded with HeLa cells were incubated without Pectinex for 60 min to allow the cells to resume growth after a medium change. Wells that contained culture medium without Pectinex served as the negative control, whereas wells with Mitomycin C (10 μg/ml) were used as the positive control. Challenge wells received 20 μl aliquots of serial dilutions of Pectinex (7.42 – 950 PGU/ml). The final volume in all the wells was titrated to 200 μl with culture medium. Experiments were performed on resting lymphocytes and neutrophils and also after stimulation with 20 μl of 0.2% w/v N-formyl-methionyl-leucyl-phenylalanine (fMLP, Sigma-Aldrich, St Louis, Missouri, USA). *f*MLP is an agent that causes lymphocytes and neutrophils to become metabolically active and mimic their behaviour as it would be in the presence of bacteria. HeLa cells were incubated for 7 days, lymphocytes for 3 days and neutrophils for 4 h.

### Viability assay of cell cultures

Cell viability was determined using the MTT (3-(4, 5-dimethylthiazol-2-yl)-2, 5-diphenyl tetrazolium bromide) assay according to Grare *et al*. [20]. A 20 μl aliquot of MTT (1 mg/ml) was added to the wells and the plate incubated for 4 h. The plates were centrifuged at 650 g for 10 min after which the supernatant was discarded. The pellet was washed with 150 μl of phosphate buffered saline (PBS) and air-dried overnight. Thereafter, 100 μl of dimethyl sulfoxide was added to each well and the plate agitated on an orbital shaker for 1 h. The optical density of the wells was measured at 570 nm (OD_570_) with a reference wavelength of 630 nm using an ELx800 Universal Microplate Reader. Percentage cell inhibition was determined using the formula:

$$ \%\ \mathrm{inhibition} = \frac{100 \times \left[\mathrm{mean}\ \mathrm{O}{\mathrm{D}}_{570}\mathrm{of}\ \mathrm{negative}\ \mathrm{control} - \mathrm{mean}\ \mathrm{O}{\mathrm{D}}_{570}\mathrm{of}\ \mathrm{sample}\right]}{\mathrm{mean}\ \mathrm{O}{\mathrm{D}}_{570}\mathrm{of}\ \mathrm{negative}\ \mathrm{control}} $$

### Polarization-optical transmitted light differential interference contrast microscopy (PlasDIC) examination of cell cultures

Cells (250 μl/well) were seeded into 24-well cell culture plates and exposed to 2-fold serial dilutions of Pectinex (7.42-950 PGU/ml). Mitomycin C (10 μg/ml) served as the positive control and untreated cells were used as negative controls. The final volume in each well was titrated to 500 μl using culture medium. Following a 24 h incubation period, cell morphology and density were observed by PlasDIC at ×400 magnification. Images were captured with AxioVision camera software.

### Microorganisms

*S. aureus* (ATCC 12600) and *P. aeruginosa* (ATCC 9027) were purchased from Davies Diagnostics and clinical strains were sourced from Department of Microbiology at the National Health Laboratory Services, Pretoria. Pure cultures were grown and maintained on Mueller-Hinton agar. Fresh 24 h sub-cultures were used for experiments.

### Minimum inhibitory and bactericidal concentrations of antibiotics in bacterial cultures

The minimum inhibitory concentrations (MIC) of of the test substances were determined using a modified broth microdilution assay [21]. Wells in 96-well microtitre plates were inoculated with 100 μl of bacterial suspension (1 × 10^6^ CFU ml^−1^), 20 μl of a serial dilution of a test agent and 80 μl of Mueller-Hinton broth. Amoxicillin-clavulanate at concentrations of 0.03 – 4.0 μg/ml^−1^ and 0.125 – 16.0 μg/ml^−1^ was used against standard and clinical strains of *S. aureus*, respectively. Ciprofloxacin (0.03 – 4.0 μg/ml^−1^) was used against both strains of *P. aeruginosa*. Pectinex (7.42 – 950 PGU/ml^−1^) was used against both bacteria. Following 24 h incubation at 37°C, 20 μl of 0.20 mg/ml^−1^*p*-INT aqueous solution (Sigma-Aldrich) was added to the wells and the cultures were incubated for a further 6 h at room temperature. The MIC was taken as the concentration of antimicrobial in the first colourless well.

The minimum bactericidal concentration (MBC) was determined by subculture of the contents of the first two clear wells obtained in the MIC assay. Plates were incubated at 37°C for 24 h. The MBC was regarded as the lowest concentration of antimicrobial agent that killed ≥ 99.9% of the initial bacterial population. Checkerboard MIC and MBC assays were used to evaluate the effects of 8 doubling concentrations of Pectinex (7.42 to 950 PGU ml^−1^) on the antibacterial efficacy across a range of 8 doubling concentrations of amoxicillin-clavulanate. The results were compared with those obtained by treatment with the antibiotic alone.

### Quantitative biofilm biomass and viability assays in bacterial cultures

Total biofilm mass and biofilm cell viability were respectively measured using a modified crystal violet (CV) assay of Pitts *et al*. [22] and a modified MTT assay of Grare *et al*. [20]. To assess the efficacy of the test agents during biofilm formation, bacterial suspensions (100 μl of standardized culture plus 80 μl of Mueller-Hinton broth) were incubated with test agents (20 μl) for 6 and 24 h at 37°C. The effects were evaluated in 6 h and 24 h biofilms by challenging the biofilms with test agents for 18 and 24 h, respectively.

To determine biofilm biomass air-dried plates were stained with 200 μl of 0.1% (w/v) CV for 30 min at room temperature after which the plates were washed thrice to remove unabsorbed stain. Plates were air-dried overnight and the stain that was adsorbed onto the biofilm was solubilized by adding 200 μl of 95% ethanol to each well (15–20 min). The OD560 nm of solubilised CV was measured with a microplate reader.

To determine cell viability following incubation, 20 μl of MTT was added to all the wells and incubated for a further 2 h at 37°C. Thereafter the plates were left to dry overnight in a dark cupboard. MTT formazan precipitate was solubilised by adding 200 μl of dimethyl sulfoxide to the wells and allowing the plates to stand in the dark for 60 min at room temperature after which OD560 nm of solubilised MTT formazan was measured. Absorbance values were corrected by subtracting the mean of absorbance readings of sterile water (experiment blanks). The percentage reductions in biofilm biomass and viable bacteria were calculated as the difference in absorbance between the treatment wells and the mean absorbance of the negative (untreated) controls and expressed as a percentage of the mean absorbance of the negative controls.

### Electron microscopy of bacterial biofilms

Biofilms were grown on glass coverslips placed in 6-well microtitre plates. During incubation, newly forming and preformed (24 h-old) biofilms were challenged for 24 h using amoxicillin-clavulanate at the MIC values (Table 1), Pectinex 118.75 PGU/ml^−1^, or a combination of the antibiotic with Pectinex. Positive (amoxicillin-clavulanate 32 μg/ml^−1^ or ciprofloxacin 8 μg/ml^−1^, and pectinex 950 PGU/ml^−1^) and negative (untreated) controls were included. Pectinex 118.75 PGU/ml was chosen as the highest non-toxic dose on the basis of the results of the cytotoxicity MTT assays and PlasDIC microscopy. After incubation the coverslips were rinsed three times with PBS. Biofilms adherent to the coverslips were prepared for SEM using the described method of Kim *et al*. [23] with modifications. Specimens were fixed in 2.5% glutaraldehyde in 0.075 M PBS for 1 h at room temperature. Secondary fixation was done with 0.5% aqueous osmium tetroxide for 1–2 h at room temperature, followed by a triple sequence of rinsing in PBS. Fixed biofilm specimens were dehydrated by transfer through increasing concentrations of ethanol (30, 50, 70, 90, 100, 100 and 100%) for 10 min at each concentration. Final preparation entailed critical point drying with liquid CO_2_ and sputter coating with gold (Emitech K550X Sputter Coater). Biofilms were imaged by scanning electron microscopy and characterized as weak, moderately adherent or fully established as described by Smith *et al*. [24].

**Table 1:** **MIC and MBC of antibiotics alone, and in antibiotic-Pectinex combinations (checkerboard), against** ***S. aureus*** **and** ***P. aeruginosa***

	**Amoxicillin-clavulanate (μg/ml)**		**Ciprofloxacin (μg/ml)**	
	***S. aureus*** **ATCC**	***S. aureus*** **clinical strain**	***P. aeruginosa*** **ATCC**	***P. aeruginosa*** **clinical strain**
MIC	0.42 (±0.08)	2.0 (±0.0)	0.13 (±0.0)	1.0 (±0.0)
MBC	0.50 (±0.0)	2.0 (±0.0)	0.33 (±0.08)	1.67 (±0.33)
Checkerboard MIC	0.42 (±0.04)	2.56 (±0.38)	0.12 (±0.01)	1.89 (±0.11)
Checkerboard MBC	0.56 (±0.09)	2.56 (±0.38)	0.32 (±0.05)	3.22 (±0.40)

[Results expressed as mean ± SEM].

### Statistical analysis

For cytotoxicity, 6 replicate experiments were performed for each cell type. Results were expressed as the percentage cell inhibition ± the standard error of the mean (SEM). The concentration that achieved 50% inhibition of cell growth (IC_50_) was determined using ED50plus version 1.0 software and expressed as IC_50_ ± SEM.

Microbiology experiments were carried out at least in triplicate and on three separate occasions. Biofilm growth (i.e. biofilm biomass and cell viability) was expressed as the percentage reduction of negative controls ± SEM. Therefore, 0% inhibition represented growth in untreated cultures. In treated cultures, a value > 0% indicated positive inhibition (i.e. reduced growth) and a value < 0% indicated negative inhibition (i.e. increased growth). Data were analyzed for statistical significance using GraphPad Prism version 5.0 software. Analysis of variables was carried out using Kruskal-Wallis one-way analysis of variance with Dunn’s multiple-comparison procedure. Comparisons were made between the different concentrations of Pectinex and the experiment controls; a *p*-value ≤ 0.05 was considered to be statistically significant.

## Results and discussion

### Effect of Pectinex on cell cultures

Enzymes are generally regarded as non-toxic and as a result only a few *in vitro* studies examining their cytotoxicity are found in the literature [13]. However, allergic reactions to inhaled aerosolized enzymes and contact irritation of the skin and eyes are known to be associated with either occupational or accidental exposure [25]. In view of the growing awareness of occupational and consumer safety associated with industrial enzymes, this study assessed the cytotoxicity of Pectinex Ultra SP-L, widely used in the food and beverage industry.

**Figure 1:**
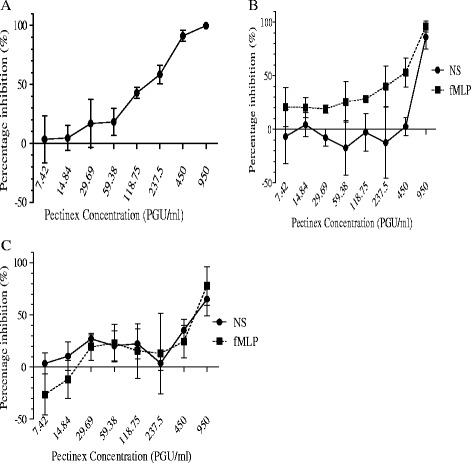
**Percentage inhibition ± SEM after Pectinex challenge in HeLa cells [A], non-stimulated (NS) and fMLP-stimulated lymphocytes [B] and neutrophils [C]**.

HeLa cell growth was significantly (p ≤ 0.05) inhibited by Pectinex concentrations ≥ 237.5 PGU/ml (Figure 1A), and the effect was comparable to mitomycin C (positive control) which inhibited cell survival by 99.0% (±1.6). The IC_50_ of Pectinex in HeLa cell cultures was 193.9 (±22.2) PGU/ml. Pectinex 950 PGU/ml caused a significant (p ≤ 0.05) decrease in the viability of both lymphocytes (Figure 1B) and neutrophils (Figure 1C). Previously, it has been reported that stimulation of blood cells with fMLP could lead to an increase in cell survival [26]. However, in this study no significant difference between the survival in fMLP-stimulated and non-stimulated cells was noted. The IC_50_ of Pectinex was 383.4 (±81.5) and 629.6 (±62.8) PGU/ml in fMLP-stimulated and non-stimulated lymphocytes, respectively, and 245.9 (±9.4) and 529.7 (±40.7) PGU/ml in fMLP-stimulated and non-stimulated neutrophils, respectively. There was no statistically significant difference in the IC_50_ between any of the cell types.

**Figure 2:**
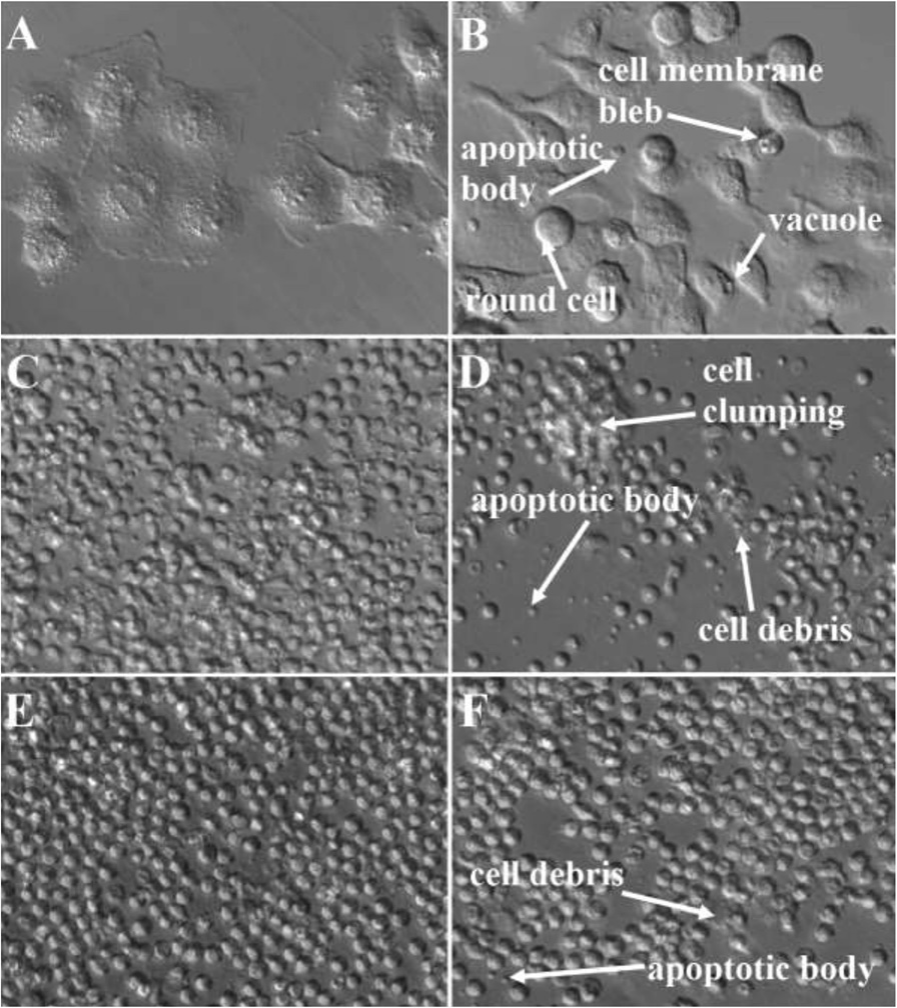
**PlasDIC microscopy images (x400 magnification) after 24 h treatment with Pectinex 237.5 PGU/ml and in untreated controls. A**: HeLa cell control; **B**: HeLa cells treated; **C**: Lymphocyte control; **D**: Lymphocytes treated; E: Neutrophil control; F: Neutrophils treated.

Examination by PlasDIC microscopy revealed morphological features characteristic of apoptosis and necrosis in all cell cultures (Figure 2). These included shrunken rounded cells, cell membrane blebs, apoptotic bodies, cytoplasmic vacuoles, cell clumping and cell debris [27]. Vacuolization and clumping (aggregation of cells and debris) were present in HeLa cells (Figure 2B) and lymphocytes (Figure 2C), respectively. In all cell cultures, Pectinex caused a dose-dependent decrease in the number of normal cell and total cell counts per microscopic field. HeLa cells (Figure 2B) and neutrophils (Figure 2 F) showed almost no evidence of morphological changes at concentrations of 7.42 – 29.69 PGU/ml and 7.42 – 118.57 PGU/ml, respectively. Conversely, lymphocytes exhibited evidence of cell death at all concentrations.

Polygalacturonase activity in Pectinex is known to catalyze the degradation of polygalacturonan in the cell walls of plants by hydrolysis of α-(1–4) glycosidic bonds that link galacturonic acid residues [28]. Studies have also reported the presence of other enzyme activity including β-galactosidase and fructosyltransferase [28,29]. In addition, there is possible protease and lipase activity in *Aspergillus*-derived enzymes [11]. In this study, the cytotoxic property of Pectinex in cell cultures was unexpected. In contrast to plant cells, mammalian cell membranes comprise phospholipids, proteins and oligosaccharides, and are susceptible to damage by proteases [30,31]. Toxicity by a non-proteolytic enzyme has been reported in a study where intraperitoneal injection of a crude fungal (*Gliomastix murorum*) enzyme extract into Swiss albino mice was found to be toxic to the liver, kidney and bone marrow [32]. This fungal extract is widely used in the food and drug industries and is known to contain an enzyme, alpha mannosidase.

From the microscopy results, it would appear that apoptosis may have been triggered as a stress response to cell membrane damage [27]. A possible mechanism for Pectinex cytotoxicity may be due to enzymatic activity on the cell membrane. Mammalian cells are covered by a carbohydrate coat (glycocalix) of membrane-bound oligosaccharides (glycolipids and glycoproteins) that are essential to the survival of cells [31,33]. Pectinex may have caused cell death by degrading the oligosaccharides thereby disrupting the function and/or integrity of the cell membrane. It is also possible that proteolytic activity may have been involved [11].

### Effect of Pectinex, amoxicillin-clavulanate and ciprofloxacin on bacteria

In a search for new ways to overcome antimicrobial resistance, the effect of Pectinex and antibiotics was studied on planktonic and biofilm cultures of standard and clinical strains of *S. aureus* and *P. aeruginosa*. These bacteria have proven ability to form biofilms under test conditions and their importance as clinical pathogens is clear [34,35]. Pectinex (7.42 – 950 PGU/ml^−1^) did not inhibit their growth in either standard or clinical cultures and therefore MIC values were not achieved. This result was consistent with the findings of Johansen *et al*. [12] who found that Pectinex (0.18 – 1800 PGU/ml^−1^) was not bactericidal. Bacterial cultures were susceptible to amoxicillin-clavulanate and ciprofloxacin (Table 1). The MIC values were within published ranges for the respective antibiotics against *S. aureus* (amoxicillin-clavulanate, 1.0 – 16.0 μg/ml) and *P. aeruginosa* (ciprofloxacin, 0.25 – 1.0 μg/ml) [36,37]. Contrary to expectations, Pectinex did not augment the bactericidal effect of either amoxicillin-clavulanate or ciprofloxacin. Instead, Pectinex-antibiotic combinations were associated with decreased susceptibility to the antibiotics in clinical cultures, as indicated by an increase in the MIC and MBC (Table 1). No change in the susceptibility to antibiotics occurred in standard cultures. In an animal model experiment, Mecikoglu *et al.* [38] showed that the proteolytic enzyme, *serratiopeptidase*, enhanced the efficacy of antibiotics in the treatment of an infected knee implant. A number of *in vitro* studies using various different antibiotic and enzyme combinations have also demonstrated increased antibiotic susceptibility in bacterial cultures [7,38,39]. Alkawash *et al*. [40] showed that alginate lyase increased the susceptibility of *P. aeruginosa* to gentamicin and alginate lyase in *P. aeruginosa* cultures. In contrast, Diaz *et al*. [41] found that the susceptibility of *P. aeruginosa* to gentamicin decreased despite treatment with alginate lyase. This suggests that different outcomes may occur even where the same combination of agents is used.

**Figure 3:**
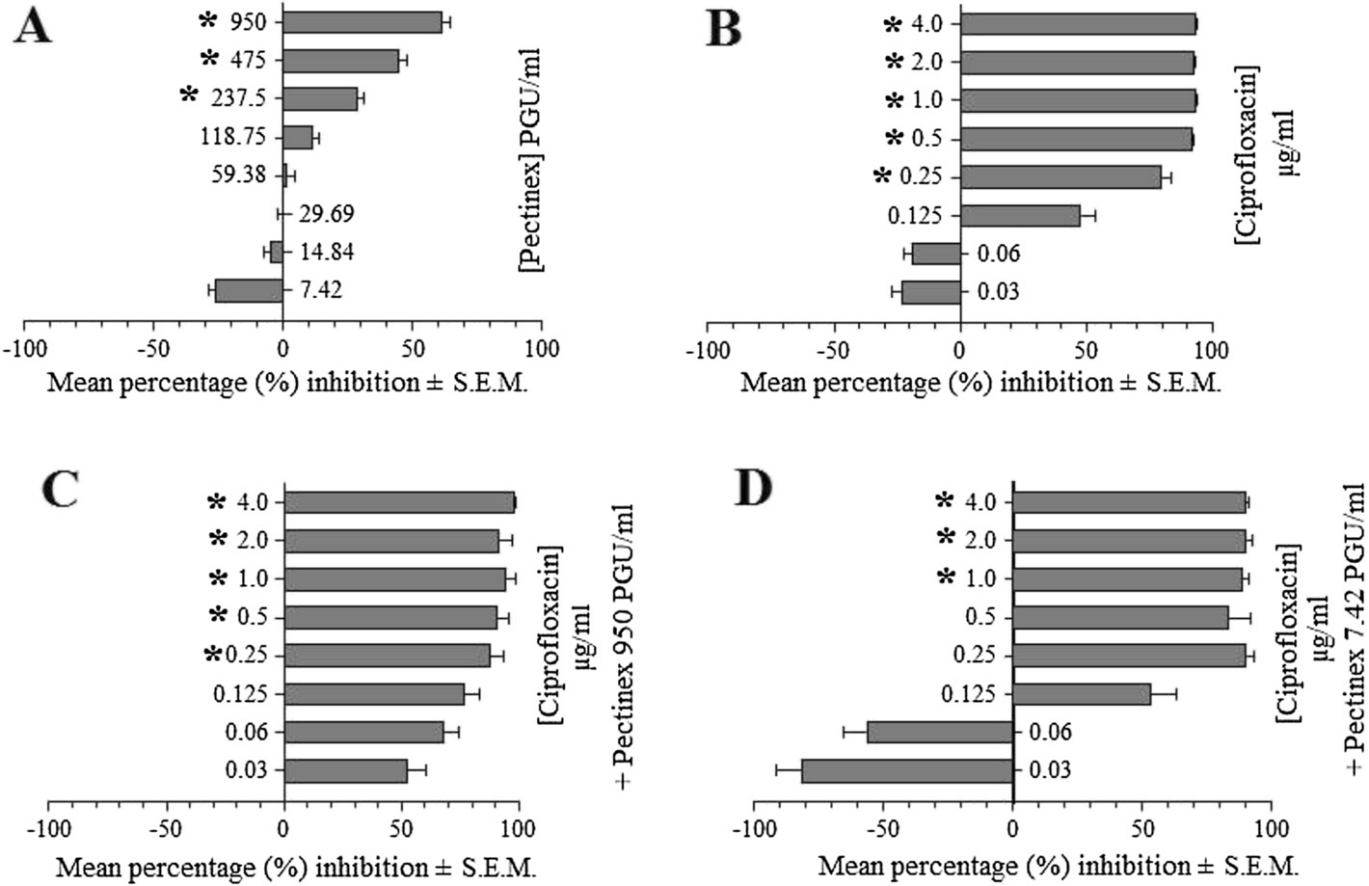
**Mean percentage (%) inhibition ± S.E.M. of biofilm formation by** ***P. aeruginosa*** ATCC strain after 6 h incubation with [A] Pectinex, [B] ciprofloxacin, [C] Pectinex 950 PGU/ml plus ciprofloxacin, and [D] Pectinex 7.42 PGU/ml plus ciprofloxacin (*indicates where p ≤ 0.05 compared to untreated controls).

This study found that low concentrations of Pectinex (≤118.57 PGU/ml^−1^) promoted biofilm adhesion, growth and cell viability in young (0 – 6 h) cultures of *S. aureus* and *P. aeruginosa*. At higher concentrations there was a mixed response of either inhibition or no effect. *P. aeruginosa* ATCC 9027 was found to be most susceptible to the antibiofilm actions of Pectinex (Figure 3A). Antibiotic concentrations ≥ MIC significantly reduced biofilm formation and cell viability in young bacterial cultures. Antibiotic concentrations below their respective MIC’s were associated with increased adhesion (Figure 3B) and cell viability. However, the findings were not statistically significant. With concurrent administration of Pectinex, biofilm biomasses and cell viabilities were statistically different (p ≤ 0.05) to untreated controls. However, no significant (p > 0.05) differences occurred between combined regimens and treatment with antibiotics alone (Figure 3 C – D). A similar pattern of responses was observed in all other tested strains with the main difference being a variation in the magnitude of the responses. Bacterial resistance to antibiotics, Pectinex and their combination increased following periods of incubation over 6 h and was found to be highest in 24 h-old preformed biofilms. A similar pattern was observed in all other tested strains, with variations in the magnitude of the responses. These findings may be explained by the fact that naturally resistant bacteria survive the initial antibiotic attack and thereafter multiply and repopulate the colonies (planktonic and biofilm) with a selection of antibiotic resistant bacteria [42]. Interestingly, Pectinex 950 PGU/ml^−1^ plus amoxicillin-clavulanate 2.0 μg/ml^−1^ inhibited *S. aureus* ATCC 12600 adhesion to a greater extent than the untreated control, although neither agent on its own produced a statistically significant effect. However, this was an isolated finding and the magnitude of change did not amount to either an additive or synergistic interaction.

The electron microscopy images of the biofilms showed that treatment with Pectinex compared to the untreated controls was associated with an increase in the number of adherent bacteria in standard cultures of *S. aureus* and *P. aeruginosa*. However, the precise responses were found to vary between the species (and strains). *S. aureus* biofilms from mature cultures (24 – 48 h) were more resistant to antimicrobial agents than the fresh (0 – 24 h) biofilms whereas the converse was true for *P. aeruginosa* biofilms. These findings were consistent with those of Leroy *et al*. [43] who found that the activity of low concentrations of Pectinex, and several other enzymes (including α-amylase, and cellulase), significantly increased bacterial adhesion in 3 h-old cultures of *Pseudoalteromonas* species, whereas higher concentrations reduced adhesion. From the evidence possible resistance mechanisms which the bacteria may have employed against Pectinex include the expression of resistance genes, the stimulation of metabolism and growth by fungal growth factors and the utilization of components of Pectinex as a source of nutrients [43,44]. In addition, the growth rate of the bacteria may have been enhanced, or the effectiveness of the enzyme diminished, by the availability of nutrient-rich growth media [45,46]. The findings do not support previous research by Johansen *et al*. [11] and Chaignon *et al*. [12], which showed Pectinex to be effective against biofilms of *P. aeruginosa* and *S. aureus*. Johansen *et al*. [11] investigated the use of Pectinex for its potential as an industrial cleaning agent and demonstrated that Pectinex concentrations as low as 0.18 – 1.8 PGU/ml effectively removed biofilms of *S. aureus. S. epidermidis, P. aeruginosa* and *P. fluorescens*.

In this study, electron microscopy images of biofilms showed monolayers of few adherent cells in the untreated controls of both bacteria. These weak biofilms may have been due to the formation of loose biofilm in the nutrient-rich culture medium. An earlier study has shown that high nutrient levels (e.g. succinate, glutamate or glucose) caused up to 80% reduction in biofilm biomass [47]. In addition, loss of biofilm cells may also have occurred during the fixation and rinsing stages of specimen preparation [23]. Nevertheless compared to the untreated controls treatment with Pectinex was associated with an increase in the number of adherent bacteria, in standard cultures of *S. aureus* and *P. aeruginosa* cultures. The responses were found to vary between the species (and strains).

**Figure 4:**
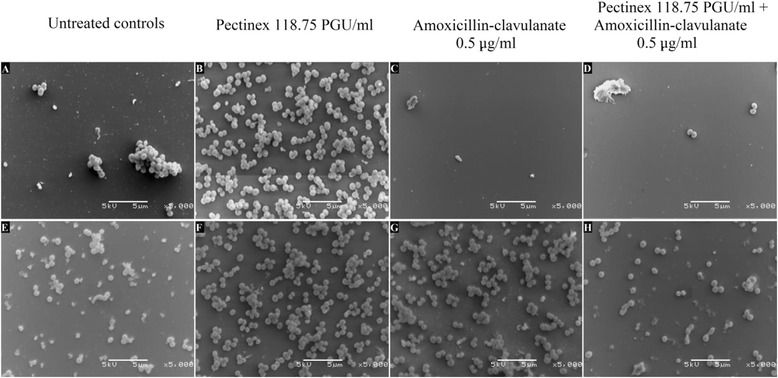
**Scanning electron microscopy (x5000 magnification) of** ***S. aureus*** **ATCC after incubation from 0 – 24 h (A – D) and 24 – 48 h (E – H).**

In *S. aureus* ATCC 12600 cultures, biofilms grew in the presence of Pectinex (Figure 4B and 4F), older biofilms (24 – 48 h) were more resistant than fresh (0 – 24 h) biofilms to the effect of the amoxicillin-clavulanate alone (Figure 4C and 4G) as well as when combined with Pectinex (Figure 4D and 4H). A similar trend was observed with biofilms from clinical cultures of *S. aureus.* Exceptionally weak biofilms were produced by both strains of *P. aeruginosa*.

## Conclusion

This study is the first to assess the cytotoxic effects of Pectinex Ultra SP-L in human cell cultures. The enzyme was found to be toxic to HeLa cells, lymphocytes and neutrophils. Apoptosis appeared to predominate following exposure to the enzyme. This suggests that the cell types are equally susceptible to Pectinex inhibition. The mechanism by which Pectinex exerted its cytotoxic action requires further investigation. Although industrial food enzymes are regarded as safe, the study supports the notion that caution should be taken during non-conventional use and exposure to these enzymes.

Pectinex was not effective in the treatment of planktonic or biofilm forms of *S. aureus* and *P. aeruginosa*. The enzyme failed to prevent the formation of new biofilm or to reduce biomass and cell viability in preformed biofilms. Rather, low concentrations of Pectinex (≤118.75 PGU/ml) were associated with increased biofilm biomass and the number of viable bacteria. Pectinex significantly reduced the antibacterial and antibiofilm actions of amoxicillin-clavulanate and ciprofloxacin against *S. aureus* and *P. aeruginosa,* respectively. Pectinex was thus found to be an unsuitable candidate for management of either planktonic or biofilm phenotypes of *S. aureus* or *P. aeruginosa.*

## References

[1] Zucca M, Savoia D (2010). The post-antibiotic era: promising developments in the therapy of infectious diseases. Int J Biomed Sci.

[2] Arias CA, Murray BE (2009). Antibiotic-resistant bugs in the 21st century - a clinical super-challenge. N Engl J Med.

[3] Public Policy IDSA (2010). The 10 x ’20 Initiative: Pursuing a global commitment to develop 10 new antibacterial drugs by 2020. Clin Infect Dis.

[4] Kaplan JB (2010). Biofilm dispersal: mechanisms, clinical implications, and potential therapeutic uses. J Dent Res.

[5] Fux CA, Wilson S, Stoodley P (2004). Detachment characteristics and oxacillin resistance of *Staphyloccocus aureus* biofilm emboli in an *in vitro* catheter infection model. J Bacteriol.

[6] Davies DG (2003). Understanding biofilm resistance to antibacterial agents. Nat Rev Drug Discov.

[7] Nickel JC, Ruseska I, Wright JB, Costerton JW (1985). Tobramycin resistance of Pseudomonas aeruginosa cells growing as a biofilm on urinary catheter material. Antimicrob Agents Chemother.

[8] Xavier JB, Picioreanu C, Rani SA, Van Loosdrecht MCM, Stewart PS (2005). Biofilm-control strategies based on enzymic disruption of the extracellular polymeric substance matrix – a modelling study. Microbiology.

[9] Van Beilen JB, Li Z (2002). Enzyme technology: an overview. Curr Opin Biotechnol.

[10] Ward OP, Qin WM, Dhanjoon J, Ye J, Singh A (2006). Physiology and biotechnology of Aspergillus. Adv Appl Microbiol.

[11] Johansen C, Falholt P, Gram L (1997). Enzymatic removal and disinfection of bacterial biofilms. Appl Env Microbiol.

[12] Chaignon P, Sadovskaya I, Ragunath C, Ramasubbu N, Kaplan JB, Jabbouri S (2007). Susceptibility of staphylococcal biofilms to enzymatic treatments depends on their chemical composition. Appl Microbiol Biotechnol.

[13] Donelli G, Francolini I, Romoli D, Guaglianone E, Piozzi A, Ragunath C, Kaplan JB (2007). Synergistic activity of dispersin B and cefamandole nafate in inhibition of staphylococcal biofilm growth on polyurethanes. Antimicrob Agents Chemother.

[14] Basketter D, Berg N, Broekhuizen C, Fieldsend M, Kirkwood S, Kluin C, Mathieu S, Rodriguez C (2012). Enzymes in cleaning products: an overview of toxicological properties and risk assessment/management. Regul Toxicol Pharmacol.

[15] Spök A (2006). Safety regulations of food enzymes. Food Technol Biotechnol.

[16] Pariza MW, Johnson EA (2001). Evaluating the safety of microbial enzyme preparations used in food processing: update for a new century. Regul Toxicol Pharmacol.

[17] Ventola CL (2009). Off-label drug information: regulation, distribution, evaluation, and related controversies. Pharm Ther.

[18] Schrek R (1936). A method for counting the viable cells in normal and in malignant cell suspensions. Am J Cancer.

[19] Pretlow TG, Luberoff DE (1973). A new method for separating lymphocytes and granulocytes from human peripheral blood using programmed gradient sedimentation in an isokinetic gradient. Immunology.

[20] Grare M, Fontanay S, Cornil C, Finance C, Duval RE (2008). Tetrazolium salts for MIC determination in microplates: Why? Which salt to select? How?. J Microbiol Methods.

[21] Eloff JN (1998). A sensitive and quick microplate method to determine the minimal inhibitory concentration of plant extracts for bacteria. Planta Med.

[22] Pitts B, Hamilton MA, Zelver N, Stewart PS (2003). A microtiter-plate screening method for biofilm disinfection and removal. J Microbiol Methods.

[23] Kim JE, Choi NH, Kang SC (2007). Anti-listerial properties of garlic shoot juice at growth and morphology of Listeria monocytogenes. Food Control.

[24] Smith K, Perez A, Ramage G, Lappin D, Gemmell CG, Lang S (2008). Biofilm formation by Scottish clinical isolates of *Staphylococcus aureus*. J Med Microbiol.

[25] Green BJ, Beezhold DH (2011). Industrial fungal enzymes: an occupational allergen perspective. J Allergy.

[26] Orie NN, Zidek W, Tepel M (1999). Chemoattractant- and mitomycin-induced generation of reactive oxygen species in human lymphocytes: The role of calcium. Exp Physiol.

[27] Elmore S (2007). Apoptosis: a review of programmed cell death. Toxicol Pathol.

[28] Cho SW, Lee S, Shin W (2001). The x-ray structure of *Aspergillus aculeatus* polygalacturonase and a modeled structure of the polygalacturonase-octagalacturonate complex. J Mol Biol.

[29] Aslan Y, Tanrıseven A (2007). Immobilization of Pectinex Ultra SP-L to produce galactooligosaccharides. J Mol Catal B: Enzym.

[30] Montilla A, Corzo N, Olani A, Jimeno ML (2009). Identification of oligosaccharides formed during stachyose hydrolysis by Pectinex Ultra SP-L. J Agric Food Chem.

[31] Singer SJ, Nicolson GL (1971). The structure and chemistry of mammalian cell membranes. Am J Pathol.

[32] Aritajat S, Saenphet K, Srikalayanukul C (2005). The toxicity of a crude enzyme extract from *Gliomastix murorum* in Swiss Albino mice. Southeast Asian J Trop Med Public Heal.

[33] Varki A (1993). Biological roles of oligosaccharides: all of the theories are correct. Glycobiology.

[34] Fux CA, Stoodley P, Hall-Stoodley L, Costerton JW (2003). Bacterial biofilms: a diagnostic and therapeutic challenge. Expert Rev Anti Infect Ther.

[35] Costerton JW, Stewart PS, Greenberg EP (1999). Bacterial biofilms: A common cause of persistent infections. Science.

[36] Hoogkamp-Korstanje JAA (1997). *In-vitro* activities of ciprofloxacin, levofloxacin, lomefloxacin, ofloxacin, pefloxacin, sparfloxacin and trovafloxacin against gram-positive and gram-negative pathogens from respiratory tract infections. J Antimicrob Chemother.

[37] Prieto J, Aguilar L, Giménez MJ, Toro D, Gómez-Lus ML, Dal-Ré R, Balcabao IP (1998). In vitro activities of co-amoxiclav at concentrations achieved in human serum against the resistant subpopulation of heteroresistant Staphylococcus aureus: a controlled study with vancomycin. Antimicrob Agents Chemother.

[38] Mecikoglu M, Saygi B, Yildirim Y, Karadag-Saygi E, Ramadan SS, Esemenli T (2006). The effect of proteolytic enzyme serratiopeptidase in the treatment of experimental implant-related infection. J Bone Jt Surg Am.

[39] Nemoto K, Hirota K, Ono T, Murakami K, Murakami K, Nagao D, Miyake Y (2000). Effect of Varidase (Streptokinase) on biofilm formed by *Staphylococcus aureus*. Chemotherapy.

[40] Alkawash MA, Soothill JS, Schiller NL (2006). Alginate lyase enhances antibiotic killing of mucoid *Pseudomonas aeruginosa* in biofilms. APMIS.

[41] Diaz E, Haaf H, Lai A, Yadana J (2011). Role of alginate in gentamicin antibiotic susceptibility during the early stages of *Pseudomonas aeruginosa* PAO1 biofilm establishment. JEMI.

[42] Singh R, Ray P, Das A, Sharma M (2009). Role of persisters and small-colony variants in antibiotic resistance of planktonic and biofilm-associated *Staphylococcus aureus*: an in vitro study. J Med Microbiol.

[43] Leroy C, Delbarre C, Ghillebaert F, Compere C, Combes D (2008). Effects of commercial enzymes on the adhesion of a marine biofilm-forming bacterium. Biofouling.

[44] Campos-Montiel RG, Viniegra-Gonzales G (1995). Microbial bioassay of fungal compounds that stimulate the growth of a consortium of anaerobic cellulolytic bacteria. Biotechnol Tech.

[45] Nazir R, Warmink JA, Boersma H, van Elsas JD (2010). Mechanisms that promote bacterial fitness in fungal-affected soil microhabitats. FEMS Microbiol Ecol.

[46] Augustin M, Ali-Vehmas T, Atroshi F (2004). Assessment of enzymatic cleaning agents and disinfectants against bacterial biofilms. J Pharm Pharm Sci.

[47] Sauer K, Cullen MC, Rickard AH, Zeef LAH, Davies DG, Gilbert P (2004). Characterization of nutrient-induced dispersion in *Pseudomonas aeruginosa* PAO1 biofilm. J Bacteriol.

